# A rare case of an EBV-positive inflammatory follicular dendritic cell tumor of the spleen

**DOI:** 10.1093/jscr/rjae600

**Published:** 2024-09-24

**Authors:** Mira Khaldoun Eid, Ahmed Samer AlQaqaa, Ibraheem J Mohammed, Awni D Shahait

**Affiliations:** School of Medicine, The University of Jordan, Amman, Jordan; School of Medicine, The University of Jordan, Amman, Jordan; Department of Pathology, Southern Illinois Healthcare, Carbondale, IL, USA; Department of Surgery, Southern Illinois University School of Medicine, Carbondale, IL, USA

**Keywords:** EBV, inflammatory follicular dendritic cell tumor, spleen

## Abstract

Epstein–Barr virus positive inflammatory pseudotumor follicular dendritic cell sarcoma (EBV+ FDCS) is a rare indolent neoplasm that presents primarily in the spleen and liver. We display a case of EBV+ FDCS in the spleen, its clinic-pathologic properties, and treatment. Our patient was evaluated following an incidental finding of a splenic mass on imaging after a traumatic injury. Computed tomography and magnetic resonance imaging both confirmed a well-circumscribed lesion in the spleen. Consequently, the patient underwent a robotic-assisted diagnostic splenectomy. Histologic examination revealed portions of spleen with partial effacement of tissue architecture by a well-circumscribed nonencapsulated mass displaying atypical, spindled cells—positive for EBER (CISH), EBV LMP1, smooth muscle actin, and clusterin—mixed inflammatory elements, and interspersed small lymphocytes.

## Introduction

Follicular dendritic cells (FDCs) play a distinctive role in regulating humoral immune responses. Situated in the B-cell follicles of secondary lymphoid tissues, these cells capture and retain antigens through highly immunogenic immune complexes [1]. FDC sarcoma (FDCS) was first described in 1986 [2], in which four patients with cervical lymphadenopathy were found to have a primary lymph node malignancy of a nonlymphomatous origin. Further workup ascertained their origin from dendritic reticulum cells. Most cases of FDCS occur mainly in extra-nodal sites (79.4%), particularly in the liver and spleen. However, it can also originate in the abdominal, cervical, axillary, and mediastinal lymph nodes (15.1%) [3].

According to the fifth edition of the World Health Organization (WHO) Classification of hemato-lymphoid tumors [4], there are two types of FDCS: conventional and Epstein–Barr virus positive (EBV+) inflammatory FDCS. EBV+ FDCS is a low-grade malignant tumor with a better prognosis than the former. They predominate in females, with a ratio of 2.2:1, middle-aged adults, and are most commonly found in the liver and spleen, and scarcely in the lymph nodes, gastrointestinal tract, lungs, palatine, and nasopharyngeal tonsils [5, 6].

Herein, we present a rare case of EBV+ inflammatory FDC/fibroblastic reticular cell tumor of the spleen and discuss its characteristics.

## Case report

**Figure 1:**
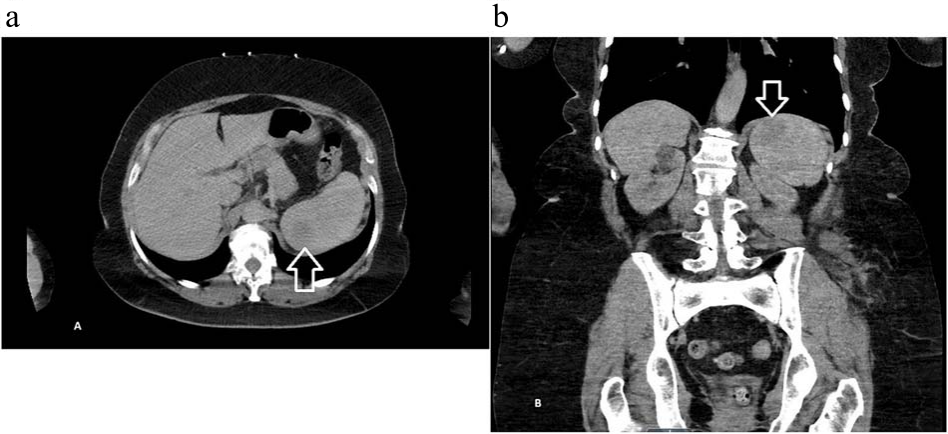
Computed tomography of the abdomen and pelvis with contrast showing an indeterminate round 3.5 cm hypo-attenuating lesion in the spleen: (a) axial and (b) coronal views with arrow marking the mass.

A 68-year-old female with a history of hypertension, gastroesophageal reflux disease, and hyperlipidemia was referred to the general surgery clinic for evaluation of a splenic mass found incidentally on a computed tomography (CT) scan performed in the emergency department, after a tree branch fell and injured her left flank. The patient was complaining from left flank pain at the time of presentation, and her physical exam showed a small nontender bruise on her left flank. Laboratory results were within normal range. CT of the abdomen and pelvis with intravenous (IV) contrast revealed an indeterminate round 3.5-cm hypoattenuating lesion in the spleen (Fig. 1).

**Figure 2:**
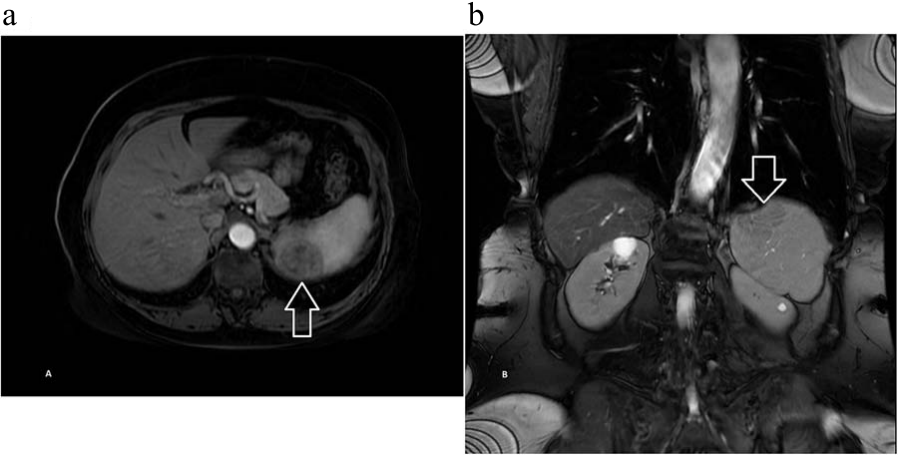
Magnetic resonance imaging of the abdomen with contrast showing a well-circumscribed 3.7 × 3.7 cm solid lesion in the lower pole of the spleen with a decreased signal on T2 on (a) axial and (b) coronal views with arrow marking the mass.

Next, magnetic resonance imaging (MRI) of the abdomen with IV contrast was obtained and revealed a well-circumscribed 3.7 × 3.7 cm solid lesion in the lower pole of the spleen with a decreased signal on T2, suggestive of atypical hemangioma versus malignancy (Fig. 2). After further discussion with the patient, she opted to undergo robotic-assisted diagnostic splenectomy. She received splenectomy vaccines 2 weeks preoperatively. The operation went smoothly, four robotic ports were utilized, and the specimen was delivered through a Pfannenstiel incision. Postoperatively, the patient’s course was complicated by rectus sheath hematoma and hemoglobin dropped to 6.7 g/dl, and she received one unit of blood with appropriate response. She was discharged home on postoperative day 3, and was doing well during her postoperative checkups.

The sample weighed 204.8 g. Sectioning unveiled a well-circumscribed solid tan mass measuring 3.7 × 3.7 × 3.5 cm, positioned subcapsular on the antihilar aspect. The remaining splenic parenchyma appeared unremarkable, devoid of identified lymph nodes.

**Figure 3:**
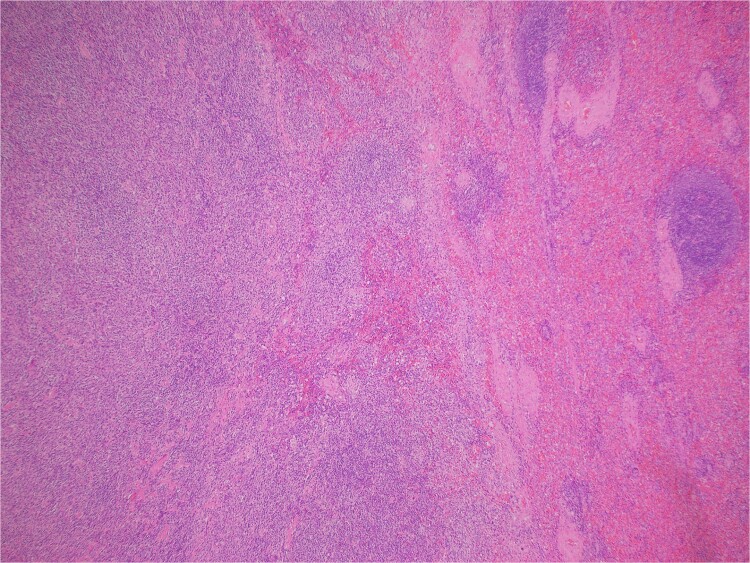
A well-circumscribed, nonencapsulated mass displaying mixed inflammatory elements (hematoxylin & eosin 40X).

**Figure 4:**
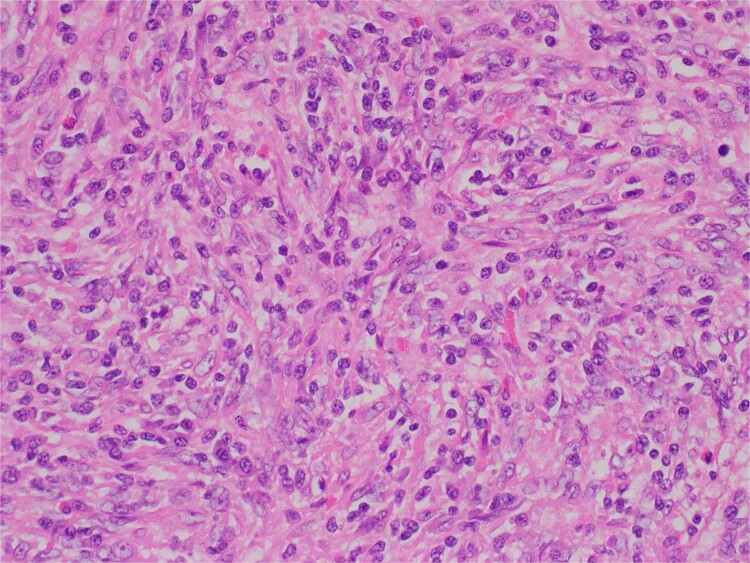
Variably increased stromal/spindled cells were noted (hematoxylin & eosin 400X).

Histologic sections revealed spleen with partial effacement of tissue architecture by a well-circumscribed, nonencapsulated mass (Fig. 3) displaying mixed inflammatory elements, including scattered reactive-appearing lymphoid cells with a vague nodular appearance, plasma cells, histiocytes, and giant cells. Variably increased stromal/spindled cells were noted throughout (Fig. 4). The background splenic architecture appeared unremarkable.

**Figure 5:**
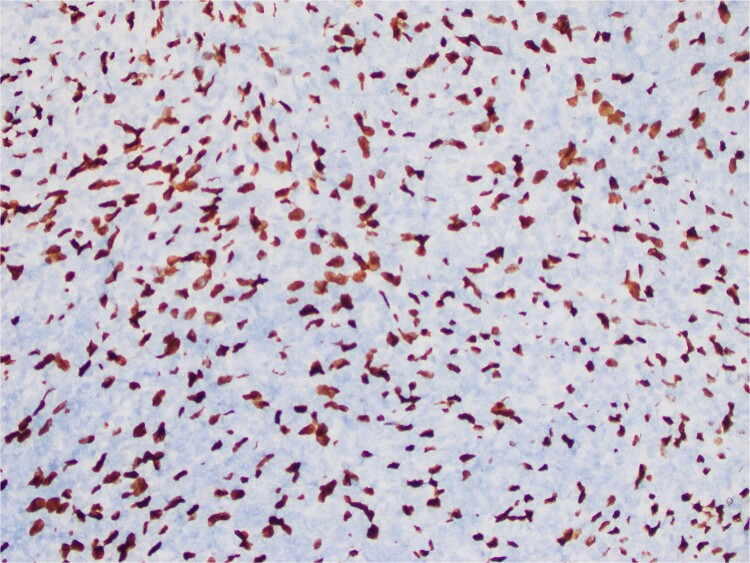
An atypical, spindled population is highlighted by CISH for EBER (EBER in situ hybridization 400X).

**Figure 6:**
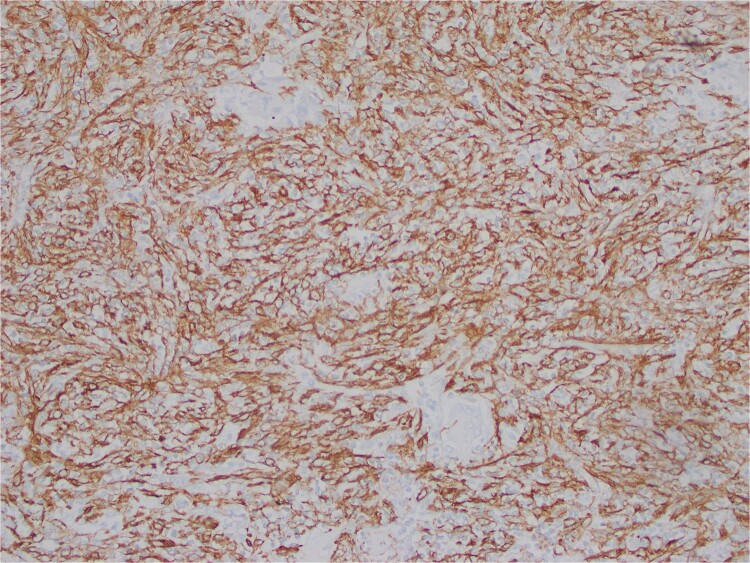
Atypical, spindled population is highlighted by clusterin (Clusterin 400X).

The interspersed lymphoid elements comprised of relatively B-cell rich nodules and numerous background T-cells. An atypical, spindled population is highlighted by chromogenic in-situ hybridization (CISH) for EBV-encoded RNA (EBER) (Fig. 5), EBV Latent Membrane Protein (LMP)1, smooth muscle actin, and clusterin (Fig. 6). Ki-67 showcased interspersed germinal centers within the inflammatory lesion and in the adjacent splenic tissue. The Ki-67 proliferation rate was otherwise low. CD31 depicted variable vascular inflammatory elements. S100 displayed scattered positivity. CD68 revealed numerous histiocytic elements. Limited PU.1 staining was observed. CD138 and IgG indicated numerous interspersed plasma cells with a focal slight increase in IgG4 positivity. CD21, CD23, and CD25 illustrated a dendritic meshwork associated with interspersed follicles. CD4 indicated numerous inflammatory elements. CD8 highlighted light-scattered T-cells and splenic sinusoids from the background spleen. Sinusoids were absent from the inflammatory lesion. Ultra-sensitive CISH dual stain for kappa and lambda revealed polytypic B-cells and plasma cells. These findings are diagnostic of an EBV+ inflammatory follicular dendritic cell/fibroblastic reticular cell tumor, according to International Consensus Classification (2022).

## Discussion

EBV+ FDCS is a rare neoplastic entity with an estimated incidence at less than 1% of all primary benign and malignant splenic tumors [7]. Numerous theories hypothesize the possible pathogenesis of this tumor. A gain of chromosome X mutation was previously identified, suggesting chromosome X mosaicism may be attributed to the development of disease [8]. Some cases showed heavy IgG4+ plasma cell infiltration, which proposed a link with the immune dysfunction caused by EBV in the etiology of the tumor [9]. Generally, the development of EBV-associated tumors is connected to an immunosuppressed state. However, insufficient evidence suggests so with IPL-FDCS [7]. Other mutations in BCL-6 corepressor ligand and Janus Kinase were described but remain clinically insignificant [8].

While most cases are asymptomatic, some may present with a painful abdominal mass, bloating, anorexia, malaise, fatigue, fever, and weight loss [10]. Ultrasound, CT, and MRI have been used to detect this tumor [11]. However, confirmatory diagnosis primarily depends on cytological and molecular findings, and EBV+ FDCS harbors both mesenchymal and inflammatory components that could present a diagnostic challenge in differentiating it from other spindle cell tumors of the spleen [8]. They display a neoplastic proliferation of spindled cells with nuclear atypia, a vesicular chromatin pattern, and conspicuous nucleoli, FDC markers like CD21, CD23, or CD35. These cells are in a background of lympho-plasmacytic infiltrate, which could include myofibroblasts that express SMA, Vimentin, and CD68. Some cases may also express an element of eosinophilia, granulomatous reactivity, necrosis, and hemorrhage [8].

There is no definitive treatment for FDCS other than complete resection of the tumor, due to its paucity and lack of targeted therapies [12]. Luckily, the prognosis is good, as Rong Ge *et al.* have concluded in their literature review between 2 and 72 months postoperatively, there were no cases of recurrence or metastatic disease after surgical intervention [13].

## References

[1] El Shikh ME , Pitzalis C. Follicular dendritic cells in health and disease. Front Immunol 2012;3:292. 10.3389/fimmu.2012.00292. 23049531 PMC3448061

[2] Monda L , Warnke R, Rosai J. A primary lymph node malignancy with features suggestive of dendritic reticulum cell differentiation. A report of 4 cases. Am J Pathol 1986;122:562–72. 2420185 PMC1888214

[3] Facchetti F , Simbeni M, Lorenzi L. Follicular dendritic cell sarcoma. Pathologica 2021;113:316–29. 10.32074/1591-951X-331. 34837090 PMC8720404

[4] Alaggio R , Amador C, Anagnostopoulos I, et al. The 5th edition of the World Health Organization classification of haematolymphoid tumours: lymphoid neoplasms. Leukemia 2002;36:1720–48. 10.1038/s41375-022-01620-2. PMC9214472 35732829

[5] Jiang XN , Zhang Y, Xue T, et al. New Clinicopathologic scenarios of EBV+ inflammatory follicular dendritic cell sarcoma: report of 9 extrahepatosplenic cases. Am J Surg Pathol 2021;45:765–72. 10.1097/PAS.0000000000001632. 33264138

[6] Lee OZJ , Omar N, Tay JK, et al. A Clinicopathology review and update of Epstein-Barr virus-associated mesenchymal Tumors. Cancers (Basel) 2023;15:5563. 10.3390/cancers15235563. PMC10705784 38067267

[7] Van Baeten C , Van Dorpe J. Splenic Epstein-Barr virus-associated inflammatory pseudotumor. Arch Pathol Lab Med 2017;141:722–7. 10.5858/arpa.2016-0283-RS. 28447898

[8] Morales-Vargas B , Deeb K, Peker D. Clinicopathologic and molecular analysis of inflammatory pseudotumor-like follicular/fibroblastic dendritic cell sarcoma: a case report and review of literature. Turk Patoloji Derg 2021;37:266–72. 10.5146/tjpath.2021.01523. 34514557 PMC10510619

[9] Choe JY , Go H, Jeon YK, et al. Inflammatory pseudotumor-like follicular dendritic cell sarcoma of the spleen: a report of six cases with increased IgG4-positive plasma cells. Pathol Int 2013;63:245–51. 10.1111/pin.12057. 23714251

[10] Zhang BX , Chen ZH, Liu Y, et al. Inflammatory pseudotumor-like follicular dendritic cell sarcoma: a brief report of two cases. World J Gastrointest Oncol 2019;11:1231–9. 10.4251/wjgo.v11.i12.1231. 31908727 PMC6937438

[11] Chen F , Li J, Xie P. Imaging and pathological comparison of inflammatory pseudotumor-like follicular dendritic cell sarcoma of the spleen: a case report and literature review. Front Surg 2022;9:973106. 10.3389/fsurg.2022.973106. 36132202 PMC9483013

[12] Abe K , Kitago M, Matsuda S, et al. Epstein-Barr virus-associated inflammatory pseudotumor variant of follicular dendritic cell sarcoma of the liver: a case report and review of the literature. Surg Case Rep 2022;8:220. 10.1186/s40792-022-01572-w. 36484868 PMC9733763

[13] Ge R , Liu C, Yin X, et al. Clinicopathologic characteristics of inflammatory pseudotumor-like follicular dendritic cell sarcoma. Int J Clin Exp Pathol 2014;7:2421–9. 24966952 PMC4069939

